# Anti-smoking initiatives and current smoking among 19,643 adolescents in South Asia: findings from the Global Youth Tobacco Survey

**DOI:** 10.1186/1477-7517-11-8

**Published:** 2014-02-25

**Authors:** Saadiyah Rao, Syeda Kanwal Aslam, Sidra Zaheer, Kashif Shafique

**Affiliations:** 1 School of Public Health, Dow University of Health Sciences, Karachi 74200, Pakistan; 2 Institute of Health and Wellbeing, Public Health, University of Glasgow, 1-Lilybank Gardens, Glasgow G12 8RZ, UK; 3 Department of Community Medicine, Dow International Medical College, Dow University of Health Sciences, Karachi 74200, Pakistan

**Keywords:** Smoking, Adolescents, Anti-smoking initiatives, South Asia

## Abstract

**Background:**

Cigarette smoking habit usually begins in adolescence. The developing countries in South Asia like Pakistan, India, Bangladesh, and Nepal, where the largest segment of the population is comprised of adolescents, are more susceptible to smoking epidemic and its consequences. Therefore, it is important to identify the association between anti-smoking initiatives and current smoking status in order to design effective interventions to curtail the smoking epidemic in this region.

**Methods:**

This is a secondary analysis of national data from the Global Youth Tobacco Survey (GYTS) conducted in Pakistan (year 2003), India (year 2006), Bangladesh (year 2007), and Nepal (year 2007). GYTS is a school-based survey of students targeting adolescents of age 13–15 years. We examined the association of different ways of delivering anti-smoking messages with students’ current smoking status.

**Results:**

A total of 19,643 schoolchildren were included in this study. The prevalence of current smoking was 5.4% with male predominance. No exposure to school teachings, family discussions regarding smoking hazards, and anti-smoking media messages was significantly associated with current smoking among male students. Participants who were deprived of family discussion regarding smoking hazards (girls: odds ratio (OR) 1.56, 95% confidence interval (CI) 0.84–2.89, *p* value 0.152; boys: OR 1.37, 95% CI 1.04–1.80, *p* value 0.025), those who had not seen media messages (girls: OR 2.89, 95% CI 1.58–5.28, *p* value <0.001; boys: OR 1.32, 95% CI 0.91–1.88, *p* value 0.134), and those who were not taught the harmful effects of smoking at school (girls: OR 2.00, 95% CI 0.95–4.21, *p* value 0.066; boys: OR 1.89, 95% CI 1.44–2.48, *p* value <0.001) had higher odds of being current smokers after multivariate adjustment.

**Conclusion:**

School-going adolescents in South Asia (Pakistan, India, Nepal, and Bangladesh) who were not exposed to anti-tobacco media messages or were not taught about the harmful effects in school or at home had higher odds of being current smokers than their counterparts.

## Background

Evidence suggests that the cigarette smoking habit usually begins before the attainment of adulthood [1], and adolescents, in particular, are more prone to develop nicotine dependence [2]. This finding is of immense concern for countries like Pakistan, India, Bangladesh, and Nepal, where cigarette smoking is highly prevalent among adolescents [3-5] and 26%–29% of national populations are comprised of individuals aged 14 years or younger [6]. Here, the largest segment of the population encompasses school-going children who are most susceptible towards experimentation with smoking. In countries with such demographic patterns, future trends of smoking-attributable morbidity and mortality should be determined accounting for the current inclination towards cigarette smoking among adolescents [3]. The emerging smoking epidemic along with its social, economic, and health consequences needs to be controlled in order to achieve tobacco elimination among school-going teenagers.

Although several studies have evaluated the effectiveness of various tobacco control initiatives, only a few have taken into account the efficiency of such programs in smoking initiation and control among schoolchildren. Evidence suggests that the targeted approach is more efficient in controlling this tobacco pandemic [7]. It is essential to determine which initiatives or programs are most appreciated by the adolescents. In a resource-limited country, evidence is needed to prove the effectiveness of any anti-tobacco initiatives before their implementation. Moreover, it is imperative to understand the particular socio-cultural context in this region, which may influence the youth's smoking behavior and hence may eventually affect their perception and response to anti-smoking initiatives.

Although South Asia is the most densely populated region of the globe, health provisions and primary preventive efforts in particular are ignored and government-sponsored anti-smoking initiatives are scarce [8]. Unfortunately, limited research has focused on this issue in the South Asian region. Therefore, the aim of this study was to determine the association of different anti-smoking initiatives with current smoking patterns in school-going children in South Asia. We also aimed to examine any gender difference in relation to anti-smoking campaigns and its impact on smoking patterns. This will guide both amendments and formulation of health policy and legislation for tobacco control in the region.

## Methods

### South Asian region

South Asia is the most densely populated geographical region in the world which harbors about a quarter of the world's population. According to the United Nations geographical region classification, South Asia is comprised of Afghanistan, Bangladesh, Bhutan, India, Iran, Maldives, Nepal, Pakistan, and Sri Lanka. Prior to 1947, India, Pakistan, and Bangladesh were a single country—British India. Furthermore, the spoken language *Hindi* is well understood in these countries leading to the dominance of Indian television and film media in this region. These factors among various others have resulted in shared societal norms in these countries. Moreover, they face common health challenges [9].

### Study design and participants

The Global Youth Tobacco Survey (GYTS) is a globally standardized cross-sectional survey to monitor youth tobacco use and track key tobacco control indicators in order to assist countries in the design, implementation, and assessment of tobacco control initiatives. This is a secondary analysis of national data from GYTS conducted in Pakistan (year 2003), India (year 2006), Bangladesh (year 2007), and Nepal (year 2007). The GYTS was a school-based survey targeting 13- to 15-year-old students. A multistage sample design was used to obtain a representative sample of students. At the first stage, schools were selected proportional to enrollment size. At the second stage, classes were randomly chosen and all students in selected classes were eligible to participate. The country coordinators followed local procedures for obtaining consent and ethical review. Details about GYTS could be found at http://www.cdc.gov/tobacco/global/.

### Response rate

The response rates of schools and students for all countries were as follows: Pakistan (no response rates are provided from the Centers for Disease Control and Prevention (CDC) official website), India (96.7%, 82.3%), Bangladesh (100%, 88.9%), Nepal (98.0%, 96.6%), respectively.

### Questionnaire

A self-administered questionnaire containing both a standard set of survey questions (for all countries) and additional questions (country specific) was used with computer-scanable answer sheets. The GYTS questionnaire included data on the prevalence of cigarette and other tobacco use, initiation, susceptibility, perceptions, attitudes, access to tobacco products, exposure to secondhand smoke, school and media anti-smoking initiatives, advertisement, as well as basic demographic information.

We included only those variables which were identical in the four selected countries. The following questions were asked to anticipate about anti-smoking initiatives in different domains:

•Home or family: ‘Has anyone in your family discussed the harmful effects of smoking with you?’ The responses for this question were ‘Yes’ or ‘No’.

•Media: ‘During the past 30 days (one month), how many anti-smoking media messages (e.g., television, radio, billboards, posters, newspapers, magazines, movies) have you seen?’ The responses for this question were ‘A lot’, ‘Few’, or ‘None’.

•Events: ‘When you go to sports events, fairs, concerts, community events, or social gatherings, how often do you see anti-smoking messages?’ The responses for this question were ‘I never go to sports events’, ‘A lot’, ‘Sometimes’, and ‘Never’.

•School: ‘During this school year, did you discuss in any of your classes the reasons why people your age smoke?’ The responses for this question were ‘Yes’, ‘No’, and ‘Not sure’. ‘During this school year, were you taught in any of your classes about the dangers of smoking?’ The responses for this question were ‘Yes’, ‘No’, and ‘Not sure’.

•‘How long ago did you last discussed smoking and health as a part of a lesson?’ The responses for this question were ‘Never’, ‘This year’ (recoded as ‘This term’), ‘Last year’, ‘Two years ago’, ‘Three years ago’, and ‘More than three years ago’ (all recoded as ‘Previous terms’).

•The question ‘During this school year, were you taught in any of your classes about the effects of smoking like it makes your teeth yellow, causes wrinkles, or makes you smell bad?’ could not be included as it was not asked in Bangladesh.

We defined ‘current smoker’ as an adolescent who smoked cigarette on 1 or more days in the past 30 days (1 month) and ‘non-smoker’ as one who did not smoke in the past 30 days. Responses to the questions were all precoded.

### Data analysis

We obtained the dataset from the CDC website and analyzed it through SPSS version 16.0 (Chicago, IL, USA). Age was categorized into two groups (≤14 years and 15–17 years) based on UNICEF early and late adolescent age limits [6]. Association between each explanatory variable and smoking had been explored initially through chi-square. Gender-stratified odds ratios (ORs) were calculated for exposure to different anti-smoking initiatives in relation to smoking status. Gender-based stratification was done for logistic regression analysis, because the literature reports gender-based differences in smoking prevalence [10]. We used ‘complex samples’ option to carry out the analysis in SPSS for multistage cluster sampling used in GYTS, accounting for country-specific PSU, stratum, and sample weight. Binary logistic regression model was used to estimate the odds ratios for association between current smoking and anti-smoking messages and other explanatory variables. An alpha level of 0.05 was established as the criterion for statistical significance for all analyses done. Total participants were 21,327; however, 1,684 participants were excluded due to missing data for any of the following variables (smoking status, 723; family discussed, 160; anti-smoking media messages, 177; anti-smoking messages at social gatherings, 89; taught about dangers of smoking, 160; why people of respondent's age smoke, 135; last discussed about smoking as a part of lesson, 102; age, 53; and gender, 85).

## Results

The final analysis included the data of 19,643 individuals: Pakistan (3,455), India (11,157), Bangladesh (2,830), Nepal (2,201). Overall, the median age of the sample was 14 years. Of these, the percentage of males was 51.2%, 57.4%, 50.6%, and 54.2% for Bangladesh, India, Nepal, and Pakistan, respectively. Of these, 1,056 (5.4%) were current cigarette smokers and 18,587 (94.6%) were non-smokers. The male gender and age group of 15 to 17 years were significantly associated with current smoking (*p* values <0.001). In addition, no family discussion about harmful effects of cigarette smoking (*p* value <0.001), seeing *a few* anti-smoking media messages (*p* value <0.001), *sometimes* seeing anti-smoking messages at social gatherings or events (*p* value <0.001), no teachings about the dangers of smoking at school (*p* value <0.001), and having discussion about smoking in *previous terms* (*p* value <0.001) were significantly associated with current cigarette smoking. Demographic characteristics of the study sample are described in Table 1. On logistic regression (unadjusted) stratified analysis for females (Table 2), those who have seen *no* anti-smoking media messages as compared to those who have seen *a lot* (OR 2.99, 95% confidence interval (CI) 1.71–5.22, *p* value <0.001), those who have *sometimes* seen anti-smoking messages at social gatherings/events as compared to those who have seen *a lot* (OR 1.80, 95% CI 1.00–3.23, *p* value 0.047), those who were not taught dangers of smoking at school as compared to those who were taught (OR 1.94, 95% CI 1.01–3.72, *p* value 0.045), and those with whom smoking was discussed as a part of lesson in the *previous terms* as compared to those with whom it was discussed in *this term* (OR 1.88, 95% CI 1.15–3.07, *p* value 0.011) had significantly higher odds of being current smokers. Moreover, current smoking was associated with increased but non-significant odds for age (OR 1.43, 95% CI 0.74–2.76, *p* value 0.275), family discussion about harmful effects of smoking (OR 1.46, 95% CI 0.84–2.56, *p* value 0.176), and seeing *a few* anti-smoking media messages (OR 1.76, 95% CI 0.91–3.42, *p* value 0.092).

**Table 1:** Characteristics of respondents by smoking status (GYTS): Pakistan, India, Bangladesh, and Nepal

**Factor**	**Non-smoker (*n*** **= 18,587)**		**Current smoker (*n*** **= 1,056)**		***p *** **value**^**a**^
	***n***	**%**	***n***	**%**	
Gender					
Male	9,988	53.7	847	80.2	<0.001
Female	8,599	46.3	209	19.8	
Age (years)					
≤ 14	12,218	65.7	582	55.1	<0.001
15–17	6,369	34.3	474	44.9	
Family discussed about harmful effects of smoking					
Yes	11,607	62.4	602	57	<0.001
No	6,980	37.6	454	43	
Frequency of anti-smoking messages seen on media					
A lot	9,221	49.6	335	33.6	<0.001
A few	5,527	29.7	412	39	
None	3,839	20.7	289	27.4	
Frequency of anti-smoking messages seen at social gatherings					
A lot	5,044	27.1	305	28.9	<0.001
Sometimes	6,530	35.1	531	50.3	
Never	2,536	13.6	108	10.2	
I never go	4,477	24.1	112	10.6	
Taught dangers of smoking at school					
Yes	10,169	54.7	438	41.5	<0.001
No	6,625	35.6	488	46.2	
Not sure	1,793	9.6	130	12.3	
Discussed at school about why people of the respondent's age smoke					
Yes	6,998	37.6	413	39.1	0.388
No	9,220	49.6	501	47.4	
Not sure	2,369	12.7	142	7.00	
When was last discussed about smoking and health as part of a lesson					
This term	4,170	22.4	213	20.2	<0.001
Previous terms	5,611	30.2	453	42.9	
Never	8,806	47.4	390	36.9	

^a^*p* values were calculated using the chi-squared test.

**Table 2:** Univariate analysis of current smoking and associated factors

**Characteristics**	**Female**		**Male**	
	**OR (95 % CI)**	***p *** **value**	**OR (95 % CI)**	***p *** **value**
Age (years)				
≤ 14	1		1	
15–17	1.43 (0.74–2.76)	0.275	1.21 (0.81–1.78)	0.347
Family discussed about harmful effects of smoking				
Yes	1		1	
No	1.46 (0.84–2.56)	0.176	1.39 (1.04–1.88)	0.025
Frequency of anti-smoking messages seen on media				
A lot	1		1	
A few	1.76 (0.91–3.42)	0.092	1.97 (1.42–2.74)	<0.001
None	2.99 (1.71–5.22)	<0.001	1.24 (0.88–1.76)	0.210
Frequency of anti-smoking messages seen at social gatherings/events				
A lot	1	1	1	
Sometimes	1.80 (1.00–3.23)	0.047	0.89 (0.66–1.20)	0.470
Never	0.71 (0.35–1.45)	0.357	0.53 (0.35–0.80)	0.002
I never go	0.43 (0.13–1.39)	0.159	0.44 (0.29–0.66)	<0.001
Taught dangers of smoking at school				
Yes	1		1	
No	1.94 (1.01–3.72)	0.045	1.77 (1.30–2.40)	<0.001
Not sure	1.52 (0.62–3.76)	0.355	1.63 (1.10–2.42)	0.013
Discussed at school about why people of the respondent's age smoke				
Yes	1		1	
No	0.81 (0.40–1.62)	0.559	0.81 (0.62–1.06)	0.134
Not sure	1.11 (0.57–2.13)	0.755	0.94 (0.67–1.33)	0.748
When was last discussed about smoking and health as part of a lesson				
This term	1		1	1
Previous terms	1.88 (1.15–3.07)	0.011	1.66 (1.20–2.30)	0.002
Never	0.72 (0.41–1.29)	0.280	0.81 (0.58–1.12)	0.202

*OR* odds ratio (unadjusted).

Logistic regression (unadjusted) stratified analysis for males (Table 2) shows that boys who had no family discussion about harmful effects of smoking as compared to those who had discussion (OR 1.39, 95% CI 1.04–1.88, *p* value 0.025), those who had seen *a few* anti-smoking media messages as compared to those who had seen *a lot* (OR 1.97, 95% CI 1.42–2.74, *p* value <0.001), those who were not taught dangers of smoking at school as compared to those who were taught (OR 1.77, 95% CI 1.30–2.40, *p* value <0.001), and those with whom smoking was discussed as a part of lesson in the *previous terms* as compared to those with whom it was discussed in *this term* (OR 1.66, 95% CI 1.20–2.30, *p* value 0.002) had significantly higher odds of being current smokers. Moreover, current smoking was associated with increased but insignificant odds for age (OR 1.21, 95% CI 0.81–1.78, *p* value 0.347) and not seeing anti-smoking media messages (OR 1.24, 95% CI 0.88–1.76, *p* value 0.210).

Multivariate logistic regression analysis for females after adjustment for age, family discussed about harmful effects of smoking, frequency of anti-smoking messages seen on media, frequency of anti-smoking messages seen at social gatherings/events, taught dangers of smoking at school, and last discussed about smoking and health as part of a lesson (Table 3) shows that participants who have *not seen* media messages as compared to those who have seen *a lot* of them (OR 2.89, 95% CI 1.58–5.28, *p* value <0.001) had significantly higher odds of being current smokers. On the other hand, age (OR 1.52, 95% CI 0.85–2.71, *p* value 0.149), family discussion about harmful effects of smoking (OR 1.56, 95% CI 0.84–2.89, *p* value 0.152), seeing *a few* anti-smoking media messages (OR 1.45, 95% CI 0.75–2.79, *p* value 0.266), *sometimes* seeing anti-smoking messages at social gatherings (OR 1.34, 95% CI 0.67–2.66, *p* value 0.401), no teaching about dangers of smoking at school (OR 2.00, 95% CI 0.95–4.21, *p* value 0.066), and discussion about smoking in *previous terms* (OR 1.41, 95% CI 0.87–2.26, *p* value 0.156) had higher but non-significant odds of being current smokers. Similarly, multivariate logistic regression analysis after adjustment for male students shows that those with whom family did not discuss about harmful effects of smoking as compared to those with whom it was discussed (OR 1.37, 95% CI 1.04–1.80, *p* value 0.025), those who had seen *a few* anti-smoking media messages as compared to those who had seen *a lot* (OR 1.86, 95% CI 1.31–2.64, *p* value <0.001), those who were not taught about dangers of smoking at school as compared to those who were taught (OR 1.89, 95% CI 1.44–2.48, *p* value <0.001), and those with whom smoking was discussed in the *previous terms* as compared to those with whom it was discussed in *this term* (OR 1.66, 95% CI 1.23–2.26, *p* value 0.001) had higher odds of being current smokers (Table 3). On the other hand, age (OR 1.33, 95% CI 0.91–1.94, *p* value 0.130) and seeing no anti-smoking media messages (OR 1.32, 95% CI 0.91–1.88, *p* value 0.134) had higher but insignificant odds of being current smokers.

**Table 3:** Multivariate analysis of current smoking and associated factors

**Characteristics**	**Female**		**Male**	
	**OR (95 % CI)**^**a**^	***p *** **value**	**OR (95 % CI)**^**a**^	***p *** **value**
Age (years)				
≤ 14	1		1	
15–17	1.52 (0.85–2.71)	0.149	1.33 (0.91–1.94)	0.130
Family discussed about harmful effects of smoking				
Yes	1		1	
No	1.56 (0.84–2.89)	0.152	1.37 (1.04–1.80)	0.025
Frequency of anti-smoking messages seen on media				
A lot	1		1	
A few	1.45 (0.75–2.79)	0.266	1.86 (1.31–2.64)	<0.001
None	2.89 (1.58–5.28)	<0.001	1.32 (0.91–1.88)	0.134
Frequency of anti-smoking messages seen at social gatherings				
A lot	1		1	
Sometimes	1.34 (0.67–2.66)	0.401	0.78 (0.56–1.09)	0.151
Never	0.42 (0.19–0.91)	0.028	0.45 (0.27–0.72)	0.001
I never go	0.27 (0.07–0.97)	0.044	0.42 (0.27–0.62)	<0.001
Taught dangers of smoking at school				
Yes	1		1	
No	2.00 (0.95–4.21)	0.066	1.89 (1.44–2.48)	<0.001
Not sure	1.07 (0.39–2.91)	0.895	1.65 (1.08–2.52)	0.020
When was last discussed about smoking and health as part of a lesson				
This term	1		1	
Previous terms	1.41 (0.87–2.26)	0.156	1.66 (1.23–2.26)	0.001
Never	0.50 (0.27–0.93)	0.028	0.74 (0.54–1.01)	0.063

^a^Multivariate models adjusted for age, family discussed about harmful effects of smoking, anti-smoking messages seen on media, anti-smoking messages seen at social gatherings, taught dangers of smoking at school, and discussion about smoking and health as part of a lesson. *OR* odds ratio (adjusted), *CI* confidence interval.

## Discussion

In our study, the prevalence of current smoking was 5.4% in this South Asian region (Pakistan, India, Bangladesh, and Nepal) with male predominance. This estimate is lower than that in African (9, 2%), Western Pacific (6, 5%), and European regions (17, 9%) and the region of the Americas (17, 5%) and comparable with that in Eastern Mediterranean (5, 0%) and previously reported prevalence for Southeast Asia (4, 3%) [11]. The lower prevalence observed in our study could be due to underreporting of smoking by young people in this region which has been indicated by a previous study [5]. Students' lack of awareness regarding tobacco-related health risks was associated with current smoking. Direct communication of smoking hazards to adolescents at school was more strongly negatively associated with odds of current smoking behavior than family discussions regarding the same at home.

Exposure to anti-smoking media messages on television, radio, billboards, posters, newspaper, magazines, and movies was also associated with non-smoking. It has been reported that anti-smoking media messages specifically on television proved to be very effective in hindering smoking among young adolescents (12–13 years), whereas exposure to radio or outdoor advertisements did not produce any significant effect [12], but we were unable to further elaborate our results. On contrary to the exposure of anti-smoking media messages, no exposure to anti-smoking messages at social gatherings/events such as fairs, concerts, sports, and community occasions has shown a protective effect against smoking. Comparable results were observed in Somaliland GYTS survey, which proposed that the content and mode of delivery of anti-smoking messages could impact the desired results [13]. Additionally, adolescents who never go to social gatherings or events were even less likely to smoke. This might be due to the fact that students who were not socializing were getting lesser opportunity to smoke or be influenced by their peers. However, we are unable to provide any explanation of this finding due to methodological constraints, as we do not have information on their family practices and parental supervision.

The male predominance has also been observed in other studies conducted on school teenagers in this region [10]. Moreover, this difference continues at higher education levels such as colleges and universities [14] and even in the general population, irrespective of urban or rural areas of inhabitation [15]. This finding is in contrast with studies conducted outside South Asia which either observed no gender difference or found a female preponderance [16]. However, it is important to note that the effect of gender on smoking predisposition has been unclear [13] and the socio-cultural factors associated with smoking may be different among the regions. Moreover, females may be reluctant to reveal their smoking habit in our region due to cultural prohibition. In the Western countries, smoking among females has become as acceptable as in males, whereas in this region, it is still considered as an objectionable practice for females [17]. Due to strong social stigma attached with this habit, females may have underreported their smoking status.

Previous studies have also reported that family-based prevention programs were inefficient in reducing the smoking susceptibility rates among 11- to 14-year-olds, which indicates further research into the dynamics of parent–child communication about smoking issues [18]. On the contrary, school-based anti-tobacco initiatives have shown to be very effective, especially if teachers as idols are discouraged from smoking within school premises [19]. Additionally, it was observed that school-based anti-smoking programs were ineffective if teachers continued to smoke in the presence of students [20]. Although tobacco control strategies need more improved approaches like involvement of the community and renewed policy-level interventions, in countries with several other competing health priorities and resource limitations, integration of tobacco control programs in the educational system could be the best way to achieve desired results with minimal financial and infrastructural burden [21]. According to the Health Belief Model, teaching youth about the dangers of smoking may reinforce their perception about the harmful effects and would result in their reduced risk to become smokers [13]. In addition, incorporation of education about specific resistance skills would be beneficial, as adolescents often do not know how to resist peer pressure [20].

Moreover, the role of parents and family in planning anti-smoking initiatives cannot be undermined, especially for non-school-going adolescents. We also found that family discussions about smoking hazards only among males were associated with non-smoking. We emphasize that our results are generalizable only to the school-going population which constitutes only a portion of the adolescent group in the South Asian region, since school enrolment rates have been reported to be low [22].

## Strength and limitations

This study examines a number of anti-smoking initiatives in the region that could be targeted in prevention programs. The questionnaire is standardized; hence, cross-country analysis was attainable. The large sample size ensured high statistical power and precise estimates. We included national datasets; thus, the findings are fairly generalizable. Apart from the above mentioned strengths, this study has some limitations. Firstly, the data were collected through self-reporting. Therefore, participants might have misreported about the smoking status, which was not confirmed through any biomarker; however, evidence suggests that health risk behaviors are correctly and reliably reported by adolescents [23]. Secondly, there could be an element of recall bias especially about the frequency of anti-smoking messages which may have led to misclassification bias in this study; however, such misclassification is likely to be non-differential which should lead to null association between exposure to anti-smoking messages and their association with smoking. However, we observed a significant positive association which is unlikely due to the misclassification bias. Thirdly, out-of-school adolescents could not be represented through this study, which remains a major limitation. Fourthly, some of the factors have shown higher odds for both genders but were non-significant in females. This could be due to the smaller proportion of smoking females in our sample.

## Conclusion

We found that school-going adolescents, particularly males who were exposed to only a few anti-tobacco media messages or were not taught about the harmful effects in school or at home, were more likely to be current smokers than those who were. A combination of school- and home-based anti-smoking interventions may be effective in the control of adolescent smoking in this region; however, further interventional studies are required on the regional population.

## Competing interests

The authors declare that they have no competing interests.

## Authors' contributions

KS conceived the idea and supervised the study. SZ and SR carried out the statistical analyses. SK and SR drafted the manuscript. All authors designed the study, contributed to interpreting the results, and read and approved the final manuscript.
